# Superelastic Nickel–Titanium (NiTi)-Based Smart Alloys for Enhancing the Performance of Concrete Structures

**DOI:** 10.3390/ma16124333

**Published:** 2023-06-12

**Authors:** Mohammad J. Alshannag, Ali S. Alqarni, Mahmoud M. Higazey

**Affiliations:** Department of Civil Engineering, King Saud University, Riyadh 11421, Saudi Arabia; mjshanag@ksu.edu.sa (M.J.A.); mhigazey@gmail.com (M.M.H.)

**Keywords:** shape memory alloys, superelasticity, concrete structures, self-centering, self-healing, prestressing

## Abstract

Recent advances in materials science have led to the development of smart materials that can continuously adapt to different loading conditions and changing environment to meet the growing demand for smart structural systems. The unique characteristics of superelastic NiTi shape memory alloys (SMAs) have attracted the attention of structural engineers worldwide. SMAs are metallic materials that can retrieve their original shape upon exposure to various temperatures or loading/unloading conditions with minimal residual deformation. SMAs have found increasing applications in the building industry because of their high strength, high actuation and damping capacities, good durability, and superior fatigue resistance. Despite the research conducted on the structural applications of SMAs during the previous decades, the existing literature lacks reviews on their recent uses in building industry such as prestressing concrete beams, seismic strengthening of footing–column connections, and fiber-reinforced concrete. Furthermore, scarce research exists on their performance under corrosive environments, elevated temperatures, and intensive fires. Moreover, the high manufacturing cost of SMA and the lack of knowledge transfer from research to practice are the main obstacles behind their limited use in concrete structures. This paper sheds light on the latest progress made in the applications of SMA in reinforced concrete structures during the last two decades. In addition, the paper concludes with the recommendations and future opportunities associated with expanding the use of SMA in civil infrastructures.

## 1. Introduction

Designing buildings today takes more than just satisfying the requirements of functionality and load-carrying capacity. There is a vital need for designing slender, long-span structures with high adaptability to changes in temperature and loading conditions. Nevertheless, designing structural elements with the highest possible strength-to-weight ratio continues to gain more popularity in construction sector due to economic reasons. Moreover, code-designed reinforced concrete (RC) moment-resisting frames are used as resisting structures against lateral cyclic loads. They withstand the damage resulting from seismic forces, including the collapse and destruction of beam–column joints that are specified as the weakest elements in structural systems. Most of the traditional strengthening techniques used for enhancing the seismic performance of RC beam–column joints subjected to cyclic lateral loads are not able to partially or fully recover the residual displacements after unloading. A promising new way of resolving this problem is to incorporate smart systems and smart materials within the beam–column joint itself. Smart systems are defined as systems that can automatically adjust their structural characteristics with respect to different loading conditions. Smart materials are the core elements in smart systems [1], which can be integrated into smart systems and provide several functions including sensing, actuation, self-adapting, self-healing, and information processes needed for monitoring.

SMAs can adapt themselves to a wide range of loading conditions and changing environments such as thermal, seismic, and wind loads, and magnetic fields. SMAs can undergo a reversible phase transformation when exposed to a temperature change and magnetic fields, which give them an advantage for changing their shape, and for use in actuation and sensing applications. SMAs can also display superelastic properties by recovering their original shape upon unloading. Moreover, SMAs can exhibit high damping capacity, by absorbing and dissipating energy under mechanical loading. This property makes them suitable for use in vibration control systems, thus improving the seismic performance of structures. SMAs are also among the smart materials that have the capability to recover their pristine shape after a significant deformation of about 8% strain [2,3,4,5,6]. This shape recovery is due to either stress- or temperature-induced phase transformations. Owing to their distinct self-centering capability, SMAs can be used in different civil engineering applications.

**Figure 1:**
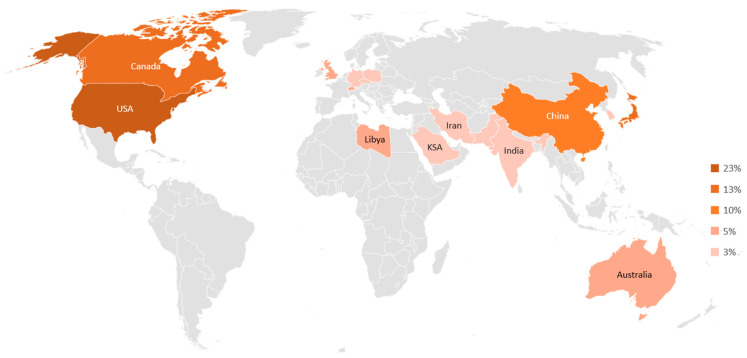
Distribution of the published research on SMAs in civil infrastructures throughout the world.

**Figure 2:**
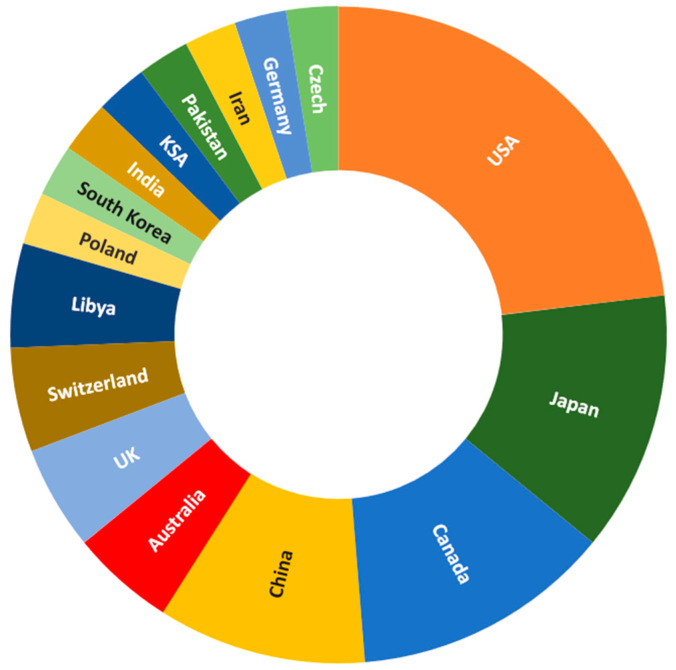
Percentage distribution of the published research on SMAs in civil infrastructures throughout the world.

SMAs were first introduced in civil engineering by Graesser and Cozzarelli [7] in the 1990s as seismic isolation materials due to their outstanding damping properties. Since then, several researchers have continued to conduct studies and present cutting-edge processing technologies to explore the distinctive characteristics of SMAs for potential applications in construction industry sector. A recent study indicated that the estimated world market size for SMA was about 11.2 billion USD in 2018 and is likely to achieve an annual growth rate of about 13% in the next 15 years [8]. Although the market share is dominated by the actuator and automotive industry, the infrastructure sector is expected to be the fastest-growing industry in the coming 5 years, with an annual growth rate of about 15% [9]. To identify the gaps in research in this area, an extensive review of the experimental studies conducted on SMAs during the last two decades is presented. Moreover, a statistical evaluation is carried out on the data gathered from more than 100 international journals indexed in web of science core collection. The statistical results are based on country of the authors, SMA distinct properties, SMA type, SMA applications, and structural elements, presented in a simple way using pie charts throughout the paper. The distribution of publications on SMA worldwide are shown in Figure 1 and Figure 2. It can be observed that about 60% of the research conducted on SMAs is shared by USA, followed by Japan, Canada, and China.

Despite the increased number of publications on SMAs during the last 5 years, they still have limited applications in various important structural elements. SMAs have mainly been used to enhance the seismic performance of the structures due to their superelastic properties. The main objective of this investigation is to present the state-of-the-art applications of SMAs under different loading conditions including monotonic, quasi-static, and reversed cyclic loadings, as well as under different environments, including elevated temperatures, intensive fires, and corrosive conditions. It provides a critical overview of the progress made in applications of NiTi SMAs in civil infrastructures during recent years, with a focus on seismic retrofitting of footing–column connections, prestressing of reinforced concrete beams, and fiber-reinforced concrete, in addition to their performance under corrosive environments and elevated temperatures. The corrosion behavior of SMA bars compared to steel bars, especially for coupled SMA–steel bars, is not yet well established in the literature. Moreover, this paper provides a summary of the properties, applications, and numerical studies of NiTi SMAs that have received limited coverage in the existing literature. The applications of SMAs covered in this paper include self-centering, retrofitting, self-healing, prestressing, and fire protection. This paper also provides detailed comparisons on the effects of SMAs in concrete structures, in terms of residual displacement, energy dissipation, and load capacity. The exact percentage increases and decreases in these properties were calculated from the published studies and presented in separate tables. The new information presented in this investigation is expected to encourage structural designers and materials scientists to explore the potential applications of smart materials for enhancing the performance of concrete structures under various loading conditions and different environments. Lastly, this paper concludes with the recommendations and opportunities for future research projects on SMA applications. It should be noted that NiTi is referred to as Nitinol SMA or simply SMA throughout this paper.

## 2. Background on SMAs and Their Distinct Properties

**Figure 3:**
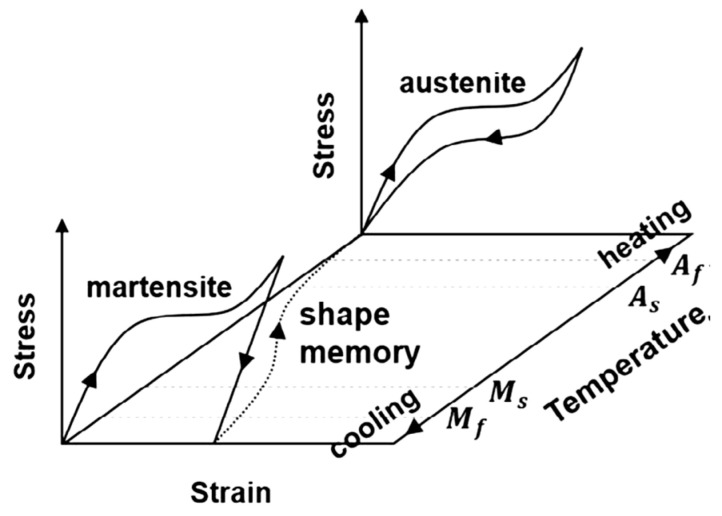
Thermomechanical behavior of SMAs. Reprinted from [11], with permission from Elsevier.

SMAs are a new group of smart materials characterized by their ability to recover large plastic strains induced while the crystal structure is in the martensitic form as shown in Figure 3. This plastic strain is recovered by raising the temperature and changing the crystal structure to the austenitic form. The alloy returns to its deformed shape once the crystal structure is transformed back to martensitic form. The speed of transformation is dependent on the speed with which the alloy can be heated. The temperature level at which martensitic transformation takes place and the shape of the hysteresis curve are dependent on the alloy composition and processing technique. When electric current is used for heating the alloy, the change can be very fast [10].

**Figure 4:**
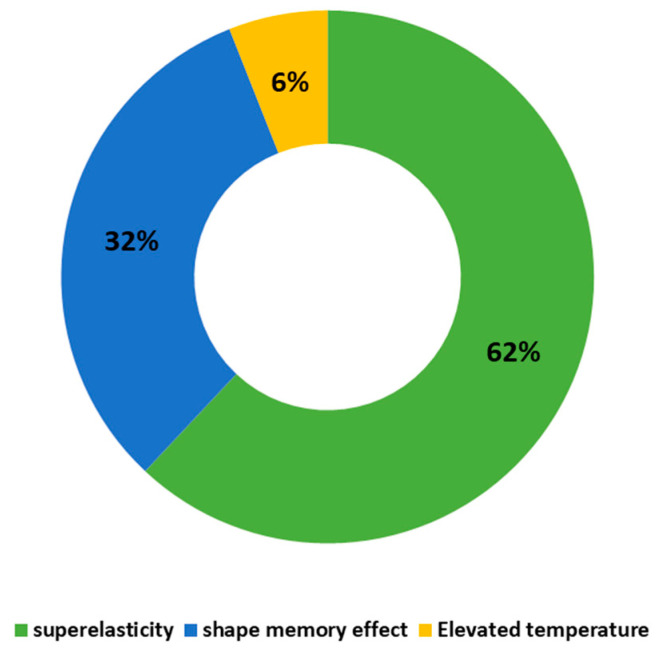
The percentage distribution of applications of SMAs based on their distinct properties.

The distinct thermomechanical properties of SMA include their shape memory effect (SME) and superelasticity effect (SE). This makes SMA an outstanding alternative for use in structures built in seismic regions. The maximum temperature that forms the start of the martensite phase and the temperature at the end of the martensite phase transformation are designated as M_s_ and M_f_, respectively, as demonstrated in Figure 3. The austenite finish temperature (A_f_) is the minimum temperature at which the superelastic effect occurs. A_s_ refers to the temperature at which the austenite phase starts. The statistical analysis shown in Figure 4 indicates that 62% of the applications of SMAs in civil infrastructures are based on their superelastic effect (SE), followed by 32% based on their shape memory effect (SME). Therefore, superelastic SMAs receive more attention in the building industry sector than those based on the shape memory effect.

### 2.1. Superelasticity of SMAs

**Figure 5:**
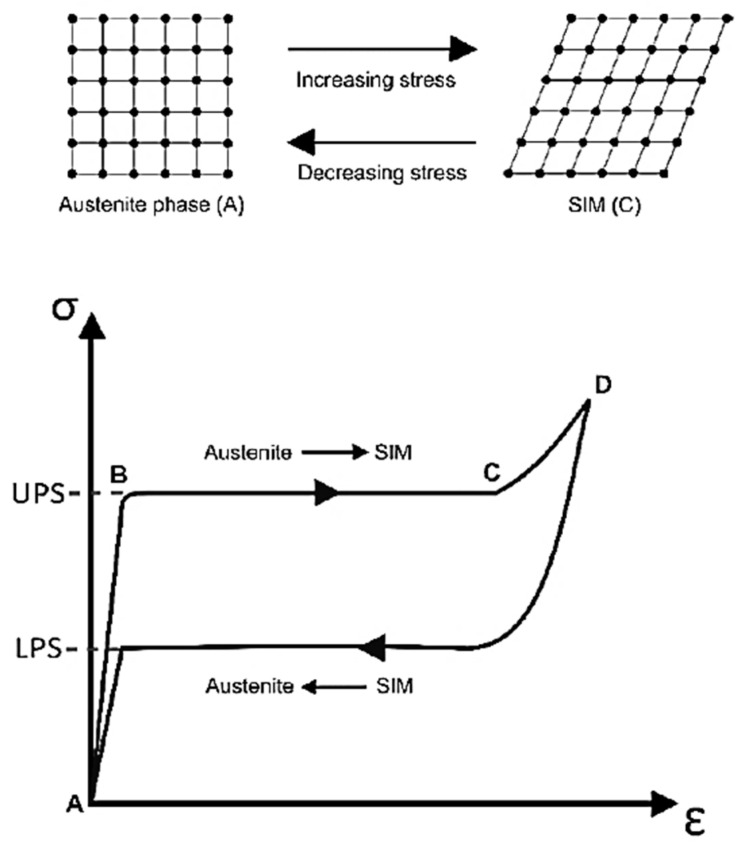
Superelasticity of SMA. Reprinted from [10], with permission from Elsevier.

**Figure 6:**
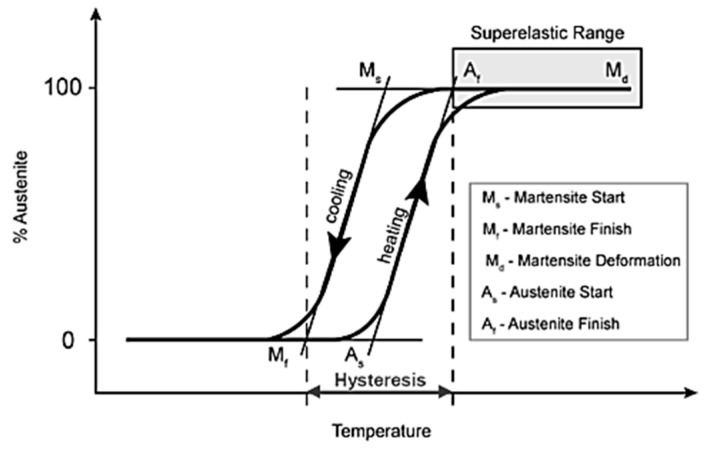
Thermal range of superelasticity of SMA. Reprinted from [11], with permission from Elsevier.

The maximum superelastic strain is the permanent strain induced by the shape memory effect of SMAs. The SMA is able to recover this strain if the temperature is above the austenite finish temperature. The superelasticity of SMAs (also known as pseudoelasticity) is determined by performing cyclic tensile testing as described in ASTM F2516 [12]. After loading the SMA specimen to 6% strain, two types of stress are identified: lower plateau stress (LPS) at 2.5% strain, and upper plateau stress (UPS) at 3% strain. The typical cyclic tensile curve presented by Khan et al. [13] for superelastic SMAs consisted of different segments (Figure 5), as demonstrated by Wu and Schetky [14] (Figure 6). Initially, it can be observed that the austenite phase displays representative elastic deformation from A to B where the UPS is reached, followed by an isostress state from B to C as the cubic austenite structure shears into detwinned SIM, and elastic deformation from C to D. After unloading from D to A, the elastic strain is recovered, and the SIM returns into the previous austenite phase. A typical Nitinol SMA displays superelasticity up to 8% strain before the onset of permanent deformation. However, depending on the SMA type, some percentage of residual deformation is there [14,15,16,17].

### 2.2. Shape Memory Effect

**Figure 7:**
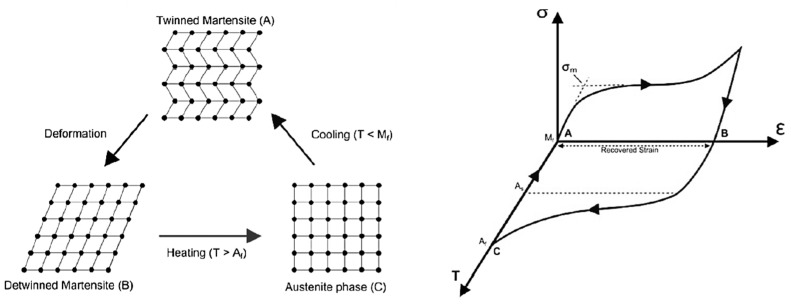
Shape memory effect of SMA. Reprinted from [10], with permission from Elsevier.

The shape memory effect, SME (also known as pseudoplasticity) is the ability of SMAs to return to their predetermined shape upon heating. The shape memory effect of SMAs is mainly induced by thermal phase transformations between martensite and austenite phases. This effect can best be illustrated using the stress–strain–temperature graph and the crystal structure presented by Wu and Schetky [14] (Figure 7). Depending on the crystal structure of the SMA, two phases can be identified: the austenite phase, which is strong and stable at high temperature; the martensite phase, which is weak and stable at low temperature. The characteristic of the transformation temperature is the hysteresis existing between the heating and cooling paths of the transformation curve shown in Figure 7. The temperature level at which the martensite transformation takes place and the shape of the hysteretic curve are dependent on the composition of the alloy and its processing technology [18].

## 3. Commonly Used SMAs

The most common type of SMAs used in civil infrastructures is NiTi (nickel–titanium), also called Nitinol alloy. This is due to the superior properties of this alloy compared to carbon steel, e.g., superelasticity, the corrosion resistance, wide range of working temperatures, and high values of strength. To date, several types of SMAs have been developed, including copper (Cu)-, niobium (Nb)-, and iron (Fe)-based SMAs [19,20,21,22]. Cu-based SMAs are alloys that contain copper as one of the main alloying elements, in addition to others. They are relatively inexpensive materials with a recoverable strain limited to 2–4% [23]. Another potential low-cost SMA type is Fe-based. Fe-based alloys have limited use in building industry applications because they are not available in large-diameter bar or wire forms. [24]. However, NiTi SMAs are widely used today, because of their excellent properties including high strength, good corrosion resistance, high fatigue life, good electrical properties, and superior SME and SE properties [25].

**Figure 8:**
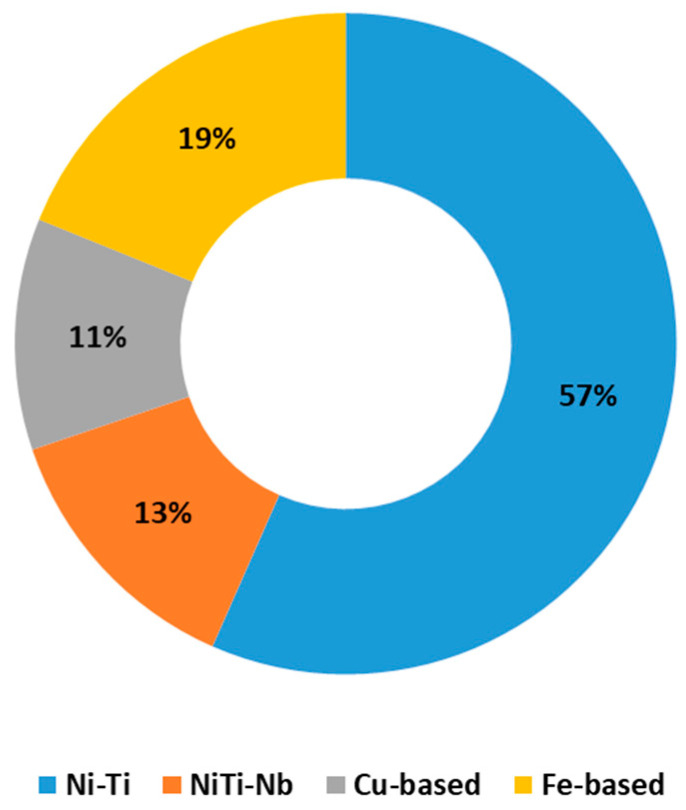
Distribution of the published research in civil engineering sorted by SMA type.

The pie chart shown in Figure 8 indicates that NiTi SMAs have been researched the most among all SMAs, and they have become the most commonly used for commercial applications. Thus, the subsequent sections focus more on Nitinol SMAs, along with their properties, performance, and applications in civil infrastructures.

## 4. Mechanical Characteristics of NiTi SMAs

The behavior of SMAs under different thermal and loading/unloading conditions is classified into two phases: martensite phase and austenite phase. Two important parameters are required in order to define the stress of an SMA and its phase: the applied strain and the working temperature [26]. Table 1 summarizes the main mechanical properties of superelastic NiTi used by researchers in civil infrastructure applications. Since the statistical analysis in Figure 4 indicated that 62% of the applications of SMA in civil infrastructures are based on their superelastic effect (SE), the superelastic response of NiTi SMAs is reviewed in depth in this paper. The dependency of this response on cyclic loading, strain rate, and working temperature is also examined in separate sections.

### 4.1. Cycling Loading

**Figure 9:**
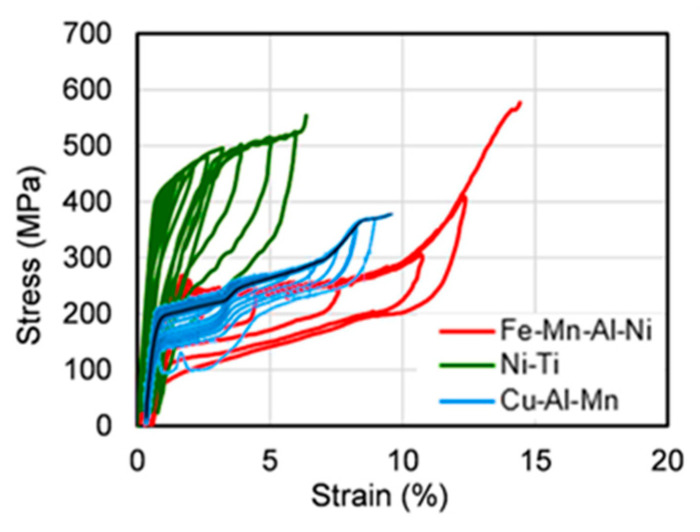
Comparison of hysteretic response of different superelastic SMAs. Reprinted from [37], with permission from Elsevier.

The superelasticity of Nitinol SMAs can be utilized effectively under lateral cyclic loads simulating earthquake loads on structures built in active seismic zones. A comparison of the hysteretic responses of three different superelastic SMAs under cyclic loads is illustrated in Figure 9 [37]. It can be seen that NiTi SMAs have good self-centering ability and high strength with adequate energy dissipation compared to copper- and iron-based SMAs.

**Figure 10:**
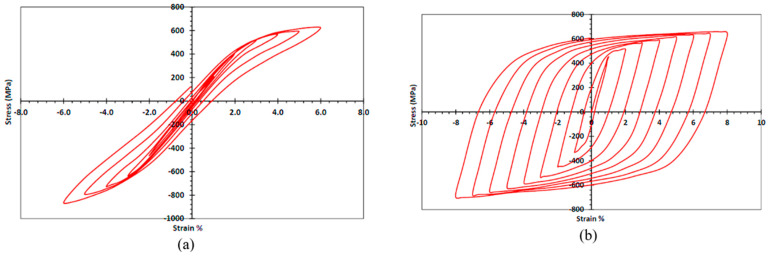
Stress–strain curve of cyclic tension–compression tests: (**a**) Nitinol SMA; (**b**) steel. Reprinted from [38], with permission from American Society of Civil Engineers.

**Figure 11:**
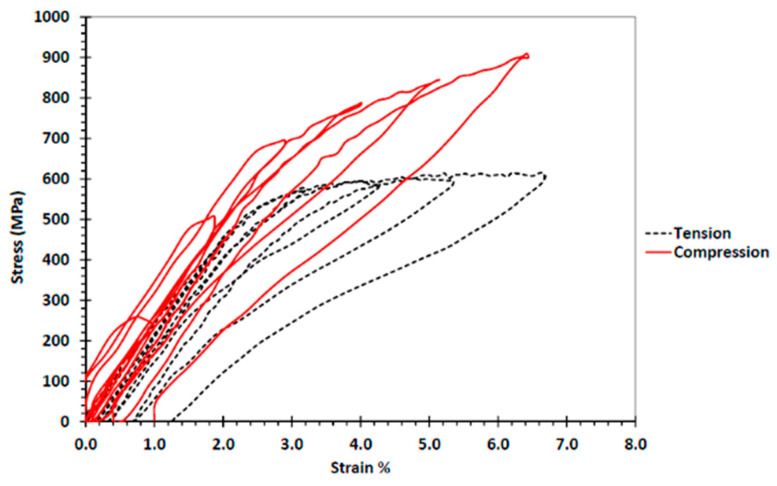
Comparison of cyclic tension and compression behaviors of Nitinol. Reprinted from [38], with permission from American Society of Civil Engineers.

Furthermore, under cyclic loading, the hysteretic response of NiTi compared to steel reinforcement is shown in Figure 10. The figure clearly shows the superior behavior of NiTi and its high capability to provide self-centering behavior compared to reinforcing steel. The superelasticity of the NiTi alloy used is almost the same in compression and tension, as shown in Figure 11 [38].

A few researchers have investigated the effect of cyclic loading on NiTi wires with a diameter of about 1–2 mm, and bars with 25.4 mm diameter [39,40,41,42,43,44,45,46]. They found that the energy dissipated decreased upon increasing the loading cycles and the material leaned toward having a stabilized performance after a specified number of cycles.

### 4.2. Strain Rate Effects

The experimental tests conducted by Azadi et al. [47] indicated that the strain rate has an important effect on the mechanical response of NiTi SMAs. Some researchers [48,49,50,51] observed that the material liberates energy as heat during the forward phase transformations, whereas it absorbs energy during unloading. They also found that the material may not have sufficient time to transfer heat to the surroundings at high strain rates. A few researchers observed a reduction in the energy dissipated with the increase in strain rate [39,42], while some other researchers [52] noticed a larger dissipation of energy at higher strain rates. Furthermore, Ozbulut and Hurlebaus [18] studied the influence of loading frequency within a common range for seismic events on the performance of SE Nitinol SMA wires. They observed up to a 47% decrease in the dissipated energy by increasing the frequency to 2 Hz. The contradiction in the results of the aforementioned studies could be due to variations of strain rates, composition of the material, and testing conditions. Saigal and Fonte [53] reported that Nitinol has an endurance limit greater than 10 million cycles at 400 MPa stress, while a small increase in stress up to 450 MPa forced the specimen to fail at 435,525 cycles (~23-fold reduction).

### 4.3. Shape Memory Effect

**Figure 12:**
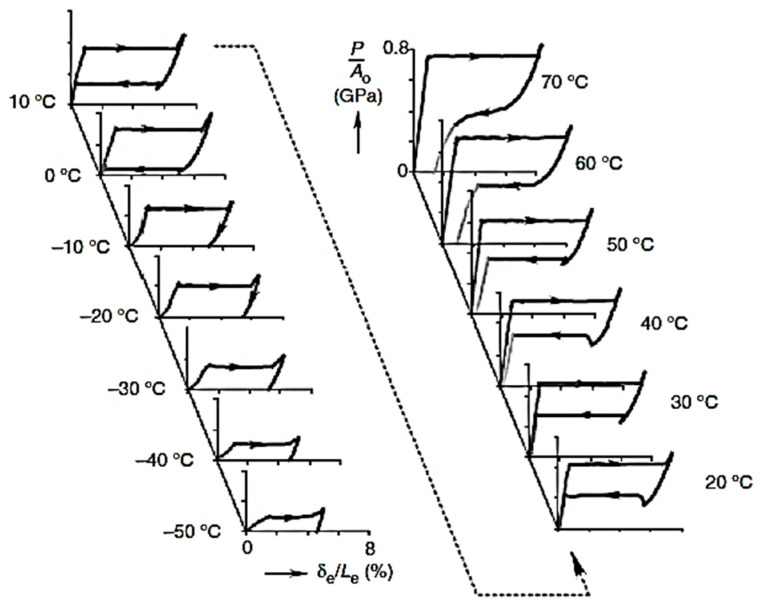
Stress–strain response of NiTi SMA at various temperatures. Reprinted from [58], with permission from Society for Experimental Mechanics.

Many researchers have investigated the effects of a change in temperature on the phase transformations and superelastic behavior of NiTi wires [18,40,54,55,56,57]. Their experimental test results revealed a significant effect of the temperature on the superelastic response of SMA. Moreover, they observed that the stress that initiates the phase transformations of SMA increases with temperature. In contrast, they found that the residual deformation and stiffness were not influenced by the temperature change in the superelastic range. A vibrant illustration of the stress–strain response of Nitinol SMA wires at different temperatures is shown in Figure 12, where the material tested exhibited a superelastic response only within 0–40 °C.

## 5. Performance of SMAs under Different Environments

Scarce information exists in the literature on the performance of SMAs under different environments. This section provides a critical review of the recent work carried out on the performance of SMAs under elevated temperatures and corrosive environments.

### 5.1. Performance of SMA under Elevated Temperatures

**Figure 13:**
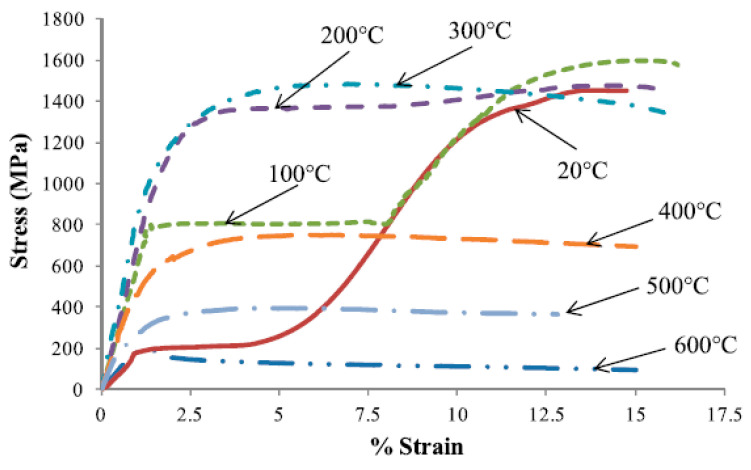
Stress–strain graphs of NiTi at elevated temperatures. Reprinted from [59], with permission from IOP Publishing.

**Figure 14:**
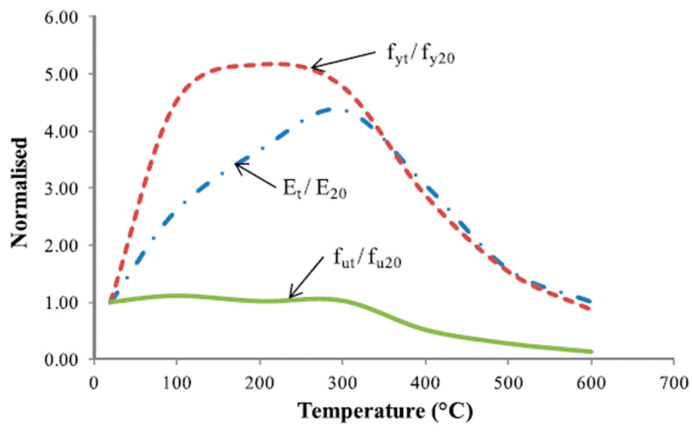
Normalized mechanical properties of NiTi alloy. Reprinted from [59], with permission from IOP Publishing.

Sadiq et al. [59] carried out some experimental tests on the effect of elevated temperatures on the stress–strain curve of NiTi SMA in tension. Unlike reinforcing steel, they found that the Young’s modulus and the yield strength of NiTi SMA increased with the increase in temperature. Moreover, the test results (Figure 13) indicated that the SMA specimens exposed to 500 °C exhibited up to a 100% increase in stress with a corresponding increase in strain of about 6% compared to the same specimens exposed to 20 °C. Furthermore, Sadiq et al. [59] found that the normalized values of Young’s modulus and yield strength shown were above 1.0 for temperatures reaching 600 °C and about 1.0 for ultimate strength at temperatures reaching 300 °C, and then dropped to 0.13 at 600 °C, as shown in Figure 14. The above results confirm that NiTi SMAs have the potential to improve the strength and resistance of RC structures against elevated temperatures and intensive fires, as discussed later in this paper.

### 5.2. Performance of SMA under Corrosive Environment

**Figure 15:**
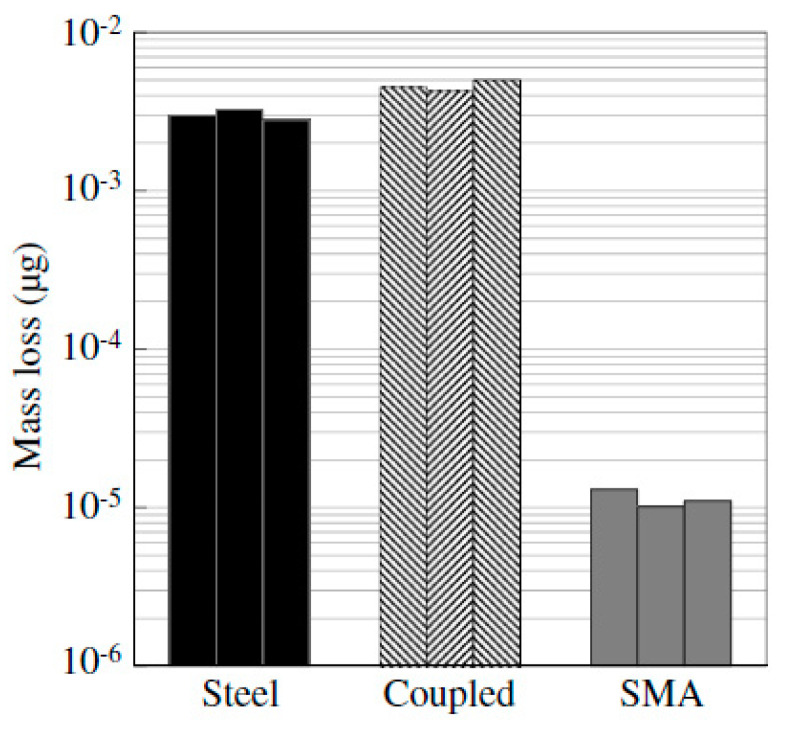
Calculated mass loss in the specimens, based on the LPR results, during 90 day exposure to the solution. Reprinted from [62], with permission from American Society of Civil Engineers.

**Figure 16:**
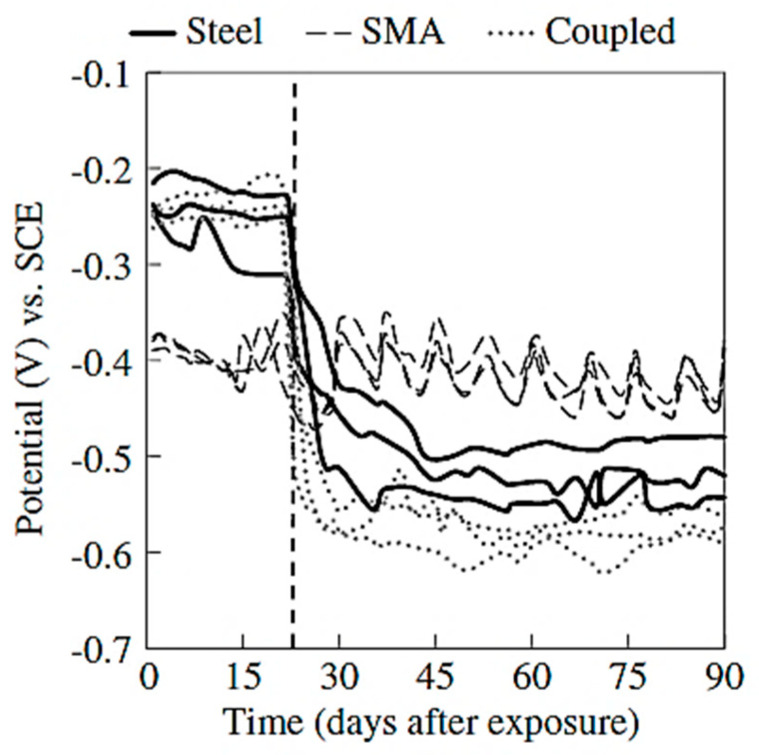
Corrosion potential values versus time; the vertical dashed line represents the date of chloride addition. Reprinted from [62], with permission from American Society of Civil Engineers.

**Figure 17:**
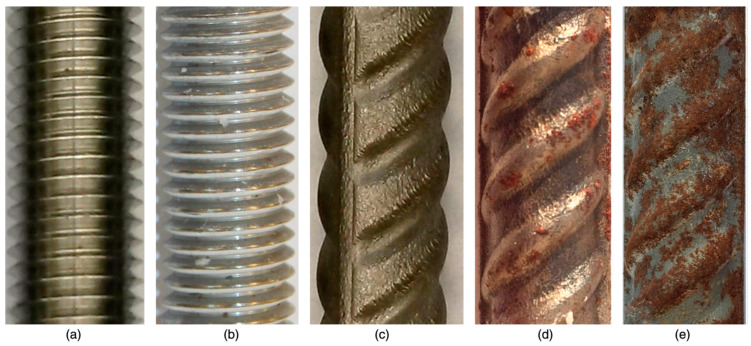
(**a**) As-received SMA; (**b**) SMA in the coupled measurement cell, 70 days; (**c**) steel before immersion in the solution; (**d**) steel specimen in the steel measurement cell, 70 days; (**e**) steel specimen in the coupled measurement cell, 70 days. Reprinted from [62], with permission from American Society of Civil Engineers.

The experimental investigations carried out by some researchers indicated that SMAs possess corrosion resistance in aggressive solutions comparable to that of carbon steel. Zhao et al. [60] found that the NiTiNb SMA was able to maintain adequate mechanical properties even after being subjected to a harsh chemical environment. Joo et al. [61] found that the corrosion resistance of an Fe-based shape memory alloy (FSMA), was about 150% more than that of steel, which had a passive coat in an alkaline environment. Furthermore, the passivated FSMA displayed a higher corrosion resistance in concrete because of its high alkalinity. Alarab et al. [62] investigated the corrosion resistance of the coupling between NiTi-based SMA and steel when immersed in a simulated concrete pore solution. Three corrosion measurements were taken for the samples: SMA alone, steel alone, and coupled SMA and steel. The specimens were submerged in a pure water solution for 20 days followed by keeping them for 70 days in a 3% chloride solution. The electrochemical measurement techniques of the corrosion resistance showed superior performance of the SMA specimens compared to steel and coupled specimens, as shown in Figure 15. However, Alarab et al. [62] observed (Figure 15) that coupling SMA with carbon steel increased the corrosion of steel significantly compared with steel-only specimens. Up to 50% more mass loss of steel was measured compared with the steel-only specimens. Alarab et al. [62] also observed from the corrosion potential values (Figure 16) that the addition of chlorides after 20 days shifted the potential values of the carbon steel and coupled specimens in the negative direction, indicating the initiation of active corrosion. Moreover, Alarab et al. [62] noticed through visual inspection of the surface of the specimens (Figure 17) no signs of corrosion for SMA specimens for both cases, i.e., coupled and uncoupled. However, some signs of corrosion products were observed on the surface of steel in coupled specimens compared with steel specimens alone, indicating that coupling increased the corrosion of steel specimens due to the galvanic effect.

## 6. Application of NiTi SMAs in Civil Infrastructures

**Figure 18:**
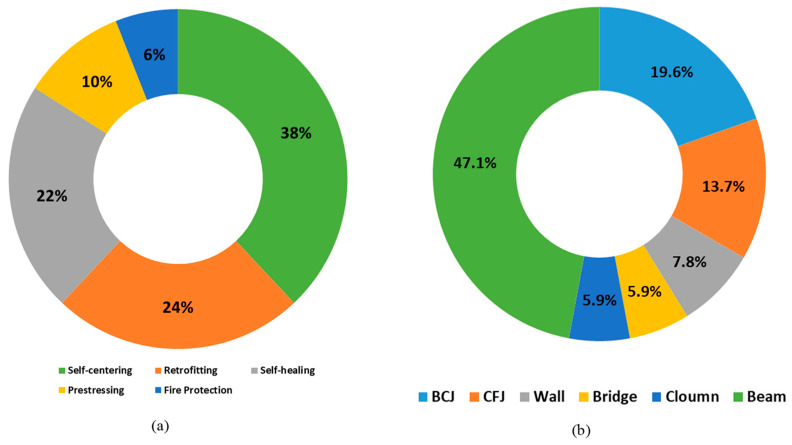
(**a**) The percentage distribution of applications based on the distinctive properties of SMAs. (**b**) The percentage distribution of the applications of SMAs based on structural components.

Although SMAs have been recognized for decades, they were not commonly used in the building sector until rather recently. Over the past two decades, some researchers presented several reviews in the literature [63,64,65,66,67,68,69,70,71,72,73,74] on applications of SMAs in civil infrastructures. The percentage distribution of applications based on the distinctive properties of SMAs is distributed among five major applications: self-centering, retrofitting, self-healing, prestressing, and fire protection, as shown in Figure 18a. The percentage distribution of applications based on the structural components is dominated by beams, followed in order by beam–column joints, column–footing joints, shear walls, bridges, and columns, as shown in Figure 18b. This section reviews the applications of superelastic SMAs in civil infrastructures because of their simplicity to use, and no need for a heat source to trigger their superelasticity. This section focuses more on the structural applications that have received little or no coverage in the recent literature.

### 6.1. Self-Centering Applications

Following the 1995 Kobe earthquake in Japan, over 100 RC columns with a residual drift ratio of over 1.75% were demolished even though they did not collapse [75]. Recently, with the increased awareness of the importance of residual displacements for post-earthquake functionality in bridges and buildings, several novel systems using SMA elements have been investigated to mitigate their effects and improve the displacement recovery of concrete structures [76,77,78,79,80,81,82,83]. These techniques are summarized in Table 2 and presented in detail in this section.

#### 6.1.1. Column–Footing Connections

**Figure 19:**
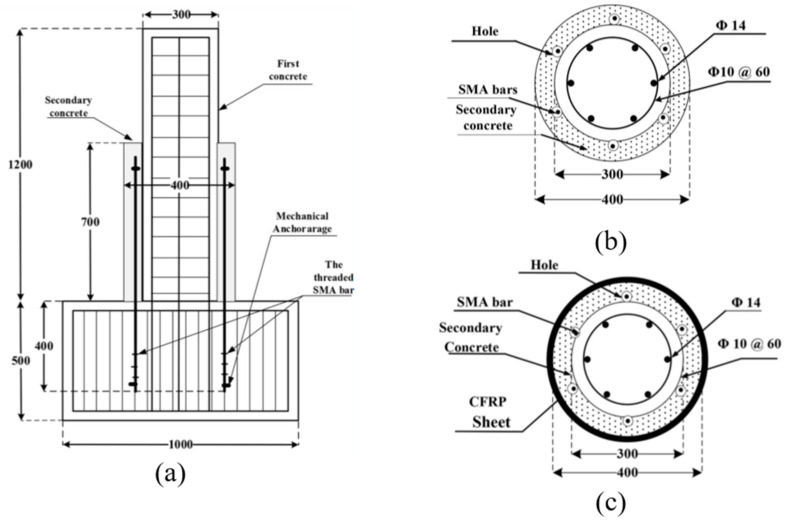
(**a**) Strengthened column–footing joint. (**b**) Reinforcement details of SMA specimen, (**c**) Reinforcement details of SMA–CFRP specimen (all dimensions are in mm). Reprinted from [34], with permission from Elsevier.

**Figure 20:**
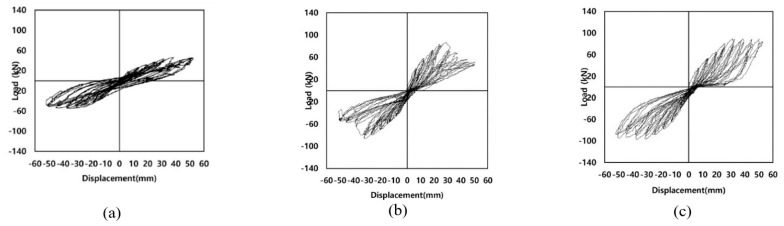
Hysteretic response of tested column–footing joint: (**a**) control specimen; (**b**) SMA specimen; (**c**) SMA–CFRP specimen. Reprinted from [34], with permission from Elsevier.

Miralami et al. [34] conducted an experimental investigation on strengthening circular RC bridge column–foundation connections using SMA bars and concrete jacketing, in addition to CFRP wrapping, as shown in Figure 19. Compared to control specimens, the specimens strengthened with the six SMA bars and wrapped with CFRP sheets, showed up to a 76% increase in lateral load capacity, up to a 43% increase in energy dissipation, recovery up to 77% of the lateral displacement, up to 82% improvement in the initial stiffness, up to 209% increase in displacement ductility, and up to 240% maximum ductility ratio, as shown in Figure 20.

**Figure 21:**
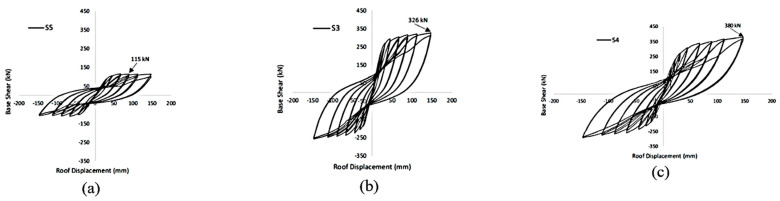
Hysteretic response of tested column–footing joint: (**a**) control specimen; (**b**) SMA–concrete specimen; (**c**) SMA–ECC specimen. Reprinted from [85], with permission from Springer.

Khan [85] presented another technique to reinforcing steel and concrete for strengthening deficient RC moment-resisting frames, using engineered cementitious composites (ECC) and shape memory alloy (SMA) bars. SMA–ECC jacketing displayed superior performance in terms of lateral load capacity and self-centering ability under repeated lateral cyclic loadings, as shown in Figure 21.

#### 6.1.2. Beam–Column Joints

**Figure 22:**
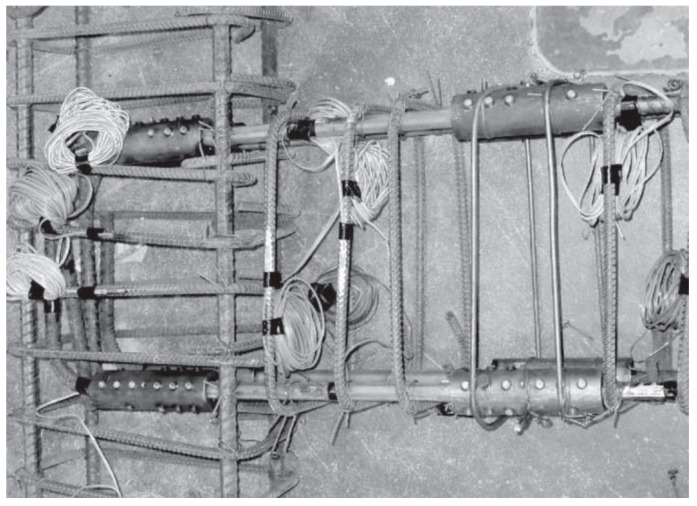
Reinforcement caging of SMA specimen. Reprinted from [33], with permission from Taylor & Francis.

**Figure 23:**
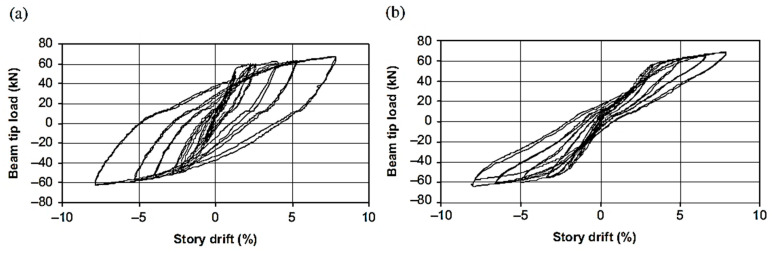
Beam load vs. story drift relationship of specimen (**a**) without SMA, and (**b**) with SMA. Reprinted from [33], with permission from Taylor and Francis.

Youssef et al. [33] tested two full-scale joint specimens to numerically and experimentally investigate the possibility of using superelastic SMA as a reinforcement for reducing the residual displacement of the joint under cyclic loading. All the investigated specimens were strengthened with a hybrid system of NiTi and steel bars, as shown in Figure 22, in addition to a control specimen reinforced with steel rebars alone. The total length of SMA rebars was 450 mm, whereas the total length of the beam was equal to 1750 mm. The results shown in Figure 23 demonstrated that SMA-reinforced beam–column joints regained a high proportion of their residual deformation, while the connection reinforced with steel rebars experienced large residual deformations. This indicates that the joint reinforced with SMA bars could remain functional after the occurrence of a severe earthquake.

**Figure 24:**
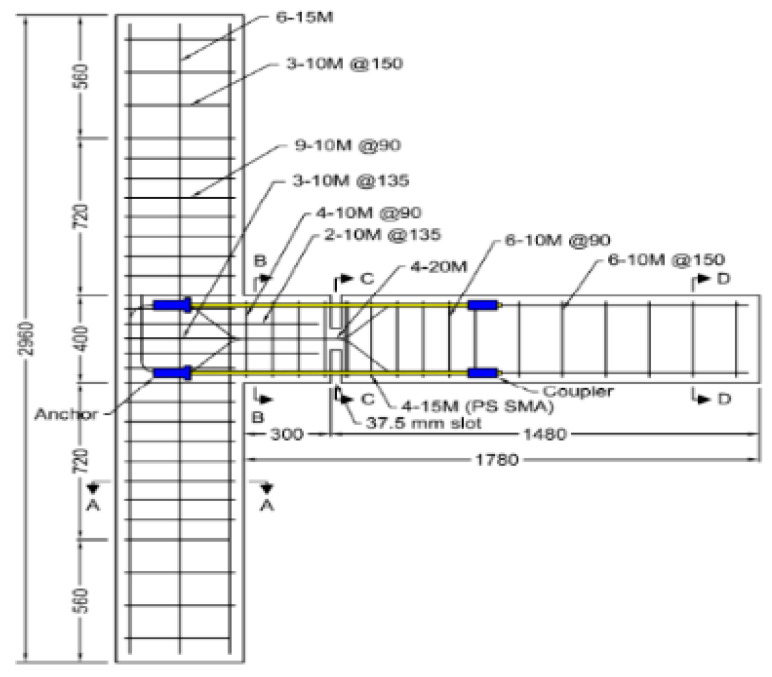
Schematic drawing of proposed plastic hinge detail. Reprinted from [32], with permission from Springer.

**Figure 25:**
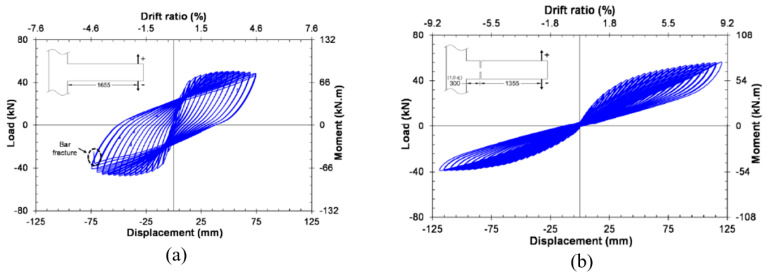
Hysteretic response of (**a**) tested control joint, and (**b**) tested SMA joint. Reprinted from [32], with permission from Springer.

Oudah and El-Hacha [32] developed a single-slotted beam technique for a self-centering RC concrete connection reinforced using SMA bars. Using this technique, the plastic hinge could be relocated away from the face of the column by slotting the beam vertically at a certain distance from the face of the column, as shown in Figure 24. The test results shown in Figure 25 proved the effectiveness of the system in mitigating the pinching shear effect, improving the self-centering capability, reducing the deformation of the joint, and relocating the plastic hinge away from the column face compared with conventional RC–BCJ connections.

#### 6.1.3. SMA Fiber Concrete

**Figure 26:**
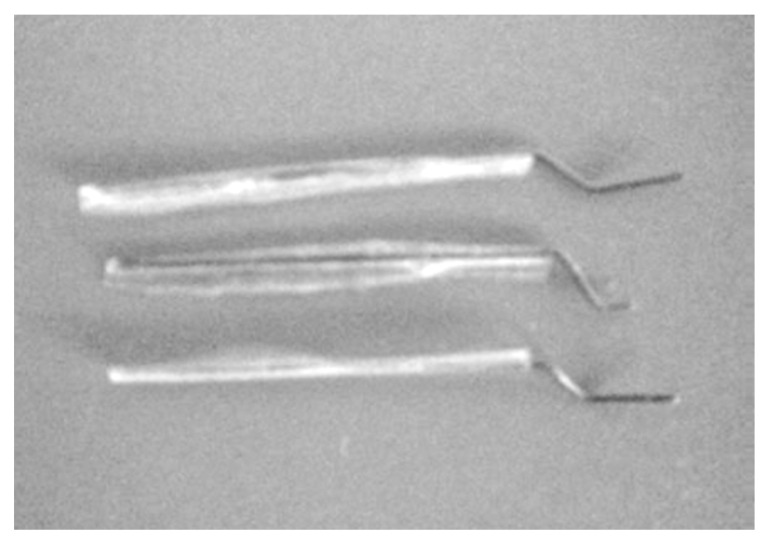
Hooked SMA fiber. Reprinted from [28], with permission from Springer.

**Figure 27:**
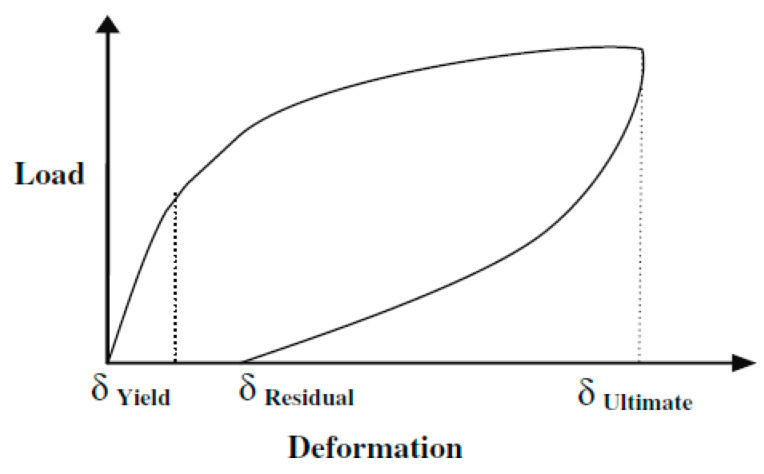
Salient points of load versus deflection behavior. Reprinted from [28], with permission from Springer.

**Figure 28:**
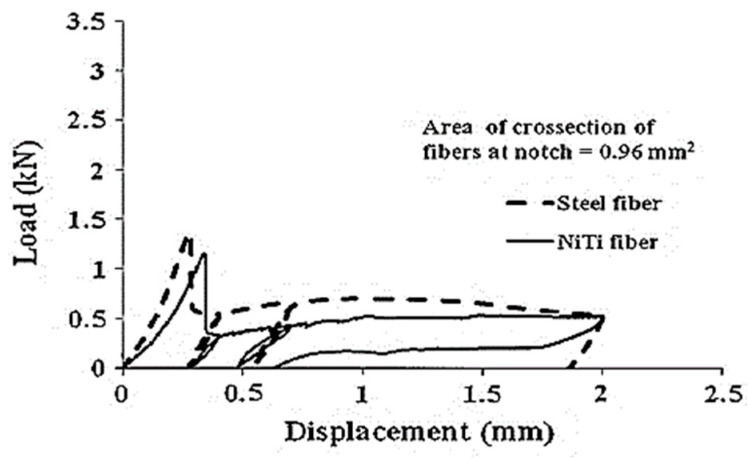
A comparison of the self-centering ability of NiTi and steel fibers having the same cross-**section** area. Reprinted from [28], with permission from Springer.

Shajil et al. [28] carried out an experimental study to estimate the self-centering capability of reinforced beams strengthened with shape memory alloy hooked fibers, as shown in Figure 26 and Figure 27, under repeated cyclic loading. The NiTi fibers used in the study had 0.5 mm diameter, 75 GPa elastic modulus, and 8% recoverable strain. According to the self-centering factor defined by the authors,

(1) $$self\;centering\;factor=\frac{\delta_{Ultimate}-\delta_{Residual}}{\delta_{Ultimate}},$$

the NiTi fiber-reinforced beam displayed a self-centering factor of 0.7, whereas the steel fiber-reinforced beam displayed a self-centering factor of 0.1. This confirmed the superior performance of NiTi fibers compared to steel fibers, as demonstrated in Figure 28.

Sherif et al. [27] performed an experimental investigation on the performance of mortar mixtures reinforced with different volume fractions of SMA fibers under cyclic loading. They found that the mortar reinforced with SMA fibers exhibited considerable improvements in ductility. Moreover, for high displacement amplitudes, they concluded that the specimens reinforced with long fibers were more effective at recovering the cracks than the short fibers. Sherif et al. [27] also observed that mortars reinforced with around 0.5% fiber content exhibited superior flexural behavior and self-centering ability compared to plain mortars.

#### 6.1.4. Shear Walls

**Figure 29:**
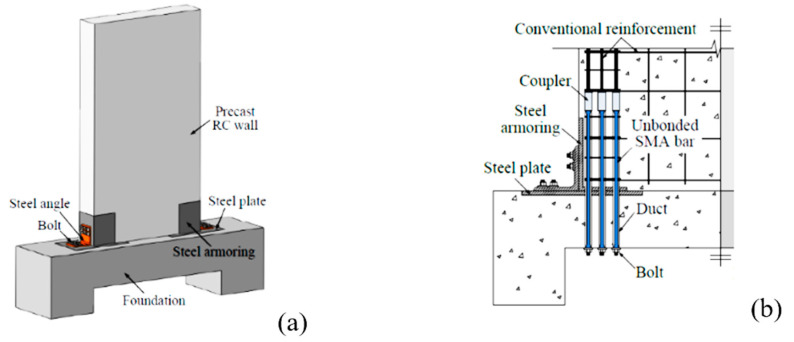
SMA precast RC wall with steel angles: (**a**) 3D view; (**b**) elevation [36]. Reprinted from [89], with permission from IOP Publishing.

**Figure 30:**
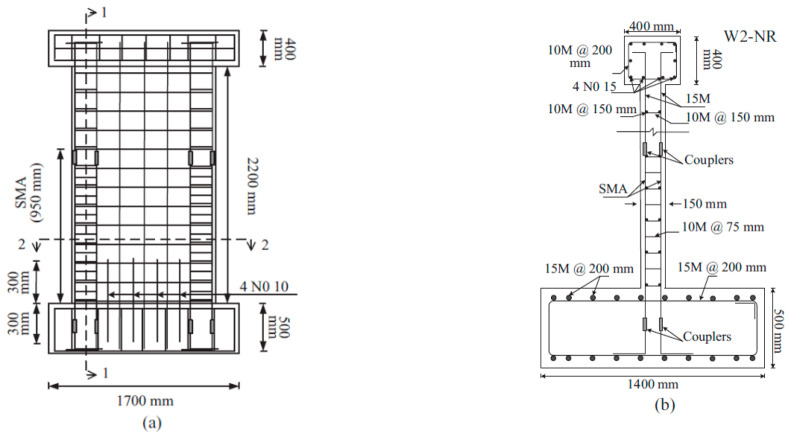
Reinforcement details of SMA shear wall: (**a**) elevation view; (**b**) section 1-1. Reprinted from [35], with permission from Elsevier.

**Figure 31:**
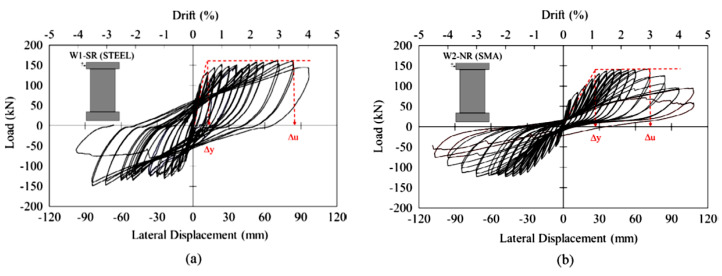
Lateral load–displacement responses: (**a**) steel shear wall; (**b**) SMA shear wall. Reprinted from [35], with permission from Elsevier.

Wang et al. [36] developed a unique precast reinforced concrete wall using SMA bars and an energy-dissipating device to enhance the seismic resistance. As shown in Figure 29, the SMA–RC walls strengthened with steel angles were found to exhibit outstanding self-centering and adequate energy dissipating capabilities. Minimal cracks were observed in the RC wall, and no yielding of the steel angles was noticed. Moreover, the energy-dissipating device can be inspected and replaced at low cost without interrupting its operation, which is highly desirable in earthquake-resistant structures. Abdulridha and Palermo [35] conducted an experimental study to assess the performance of a hybrid SMA-deformed steel reinforced concrete shear wall under reversed cyclic loading as shown in Figure 30. Their test results demonstrated that the hybrid SMA wall was more effective in recovering its self-centering ability than the steel reinforced walls, after being subjected to drifts exceeding 4%, as shown in Figure 31. The restoring capacity of the hybrid SMA wall was about 92% compared to 31% for steel-reinforced walls.

**Figure 32:**
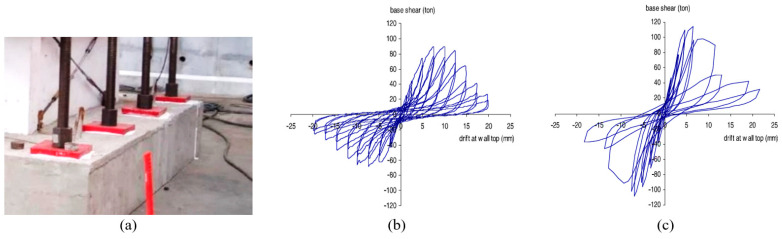
(**a**) Scheme of shear wall SMA bracing. (**b**) Hysteretic response of control wall. (**c**) Hysteretic response of retrofitted wall. Reprinted from [88], with permission from the American Society of Civil Engineers.

Effendy et al. [88] retrofitted an RC shear wall using SMA bracing subjected to high seismic loading as shown in Figure 32. The test results showed that the NiTi bracing enhanced the performance of shear wall and significantly reduced the residual displacement by about 67%, as shown in Figure 32.

#### 6.1.5. Bridges

**Figure 33:**
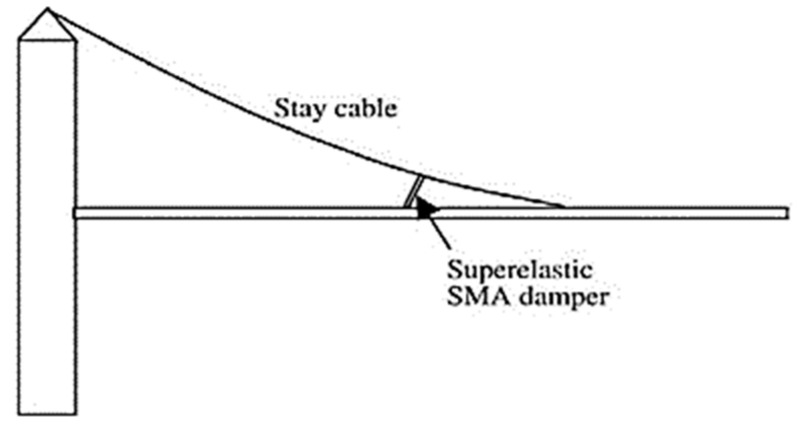
SMA damper for a stay-cable bridge. Reprinted from [10], with permission from Elsevier.

**Figure 34:**
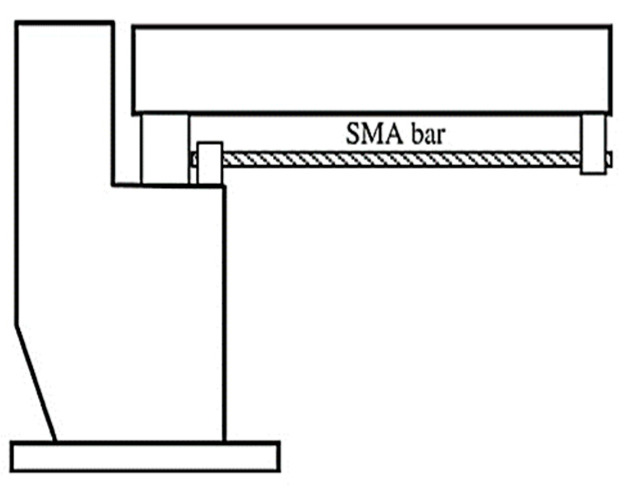
SMA restrainer for a simply supported bridge deck. Reprinted from [91], with permission from Elsevier.

**Figure 35:**
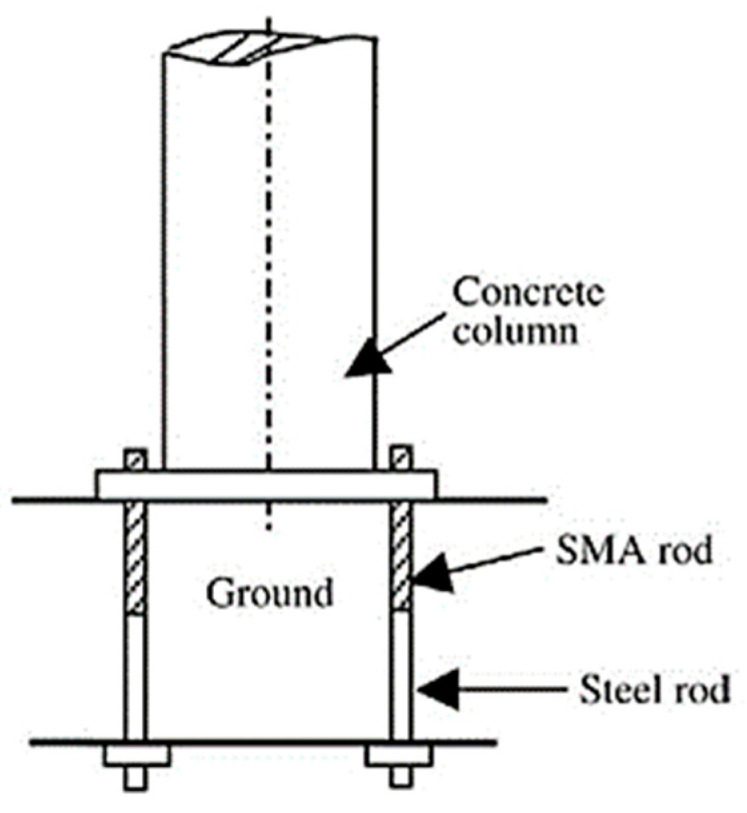
SMA bar anchorage for a structural column. Reprinted from [10], with permission from Elsevier.

The 1995 Kobe and 1994 Northridge earthquakes emphasized the significance of residual deformations in bridges and buildings, because of the huge post-maintenance costs incurred following these earthquakes. Many researchers have used SMAs in different bridge components to improve their performance and reduce the residual deformations after earthquakes. The proposed components include RC bridge columns, bridge piers, bridge decks, and girders with prestressed wires. Li et al. [90] proposed a combined cable–SMA damper system for mitigating the vibration of a stay-cable bridge, as shown in Figure 33. They concluded that the proposed superelastic SMA damper could suppress the cable’s vibration. DesRoches and Delemont [91] proposed using superelastic SMA bar restrainers for seismic retrofit of simply supported bridge decks, as shown in Figure 34. Test results indicated that the SMA restrainer provided a large elastic deformation range compared with steel restrainer cables. Tamai and Kitagawa [41] proposed an anchorage system made of Nitinol SMA rods and steel bars, as shown in Figure 35, for dissipating energy and reducing the bridge vibration under severe seismic ground motion. They concluded that the anchorage system was effective in preventing plastic deformation and damage in RC columns.

Zheng et al. [92] proposed novel SMA washer-based piers for enhancing the performance of bridges against seismic loading. SMA washers proved to be effective in providing flexibility and deformability through using different stack patterns.

**Figure 36:**
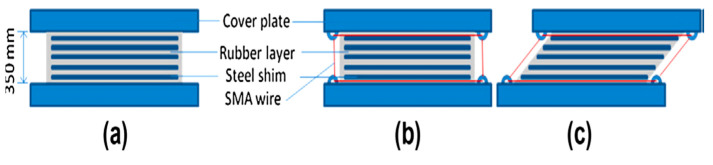
Description of the isolation bearing (pad 1000 mm × 1000 mm with 350 mm height): (**a**) HDRB, where the rubber layers are vulcanized by steel shims; (**b**) SRB in undeformed condition; (**c**) SRB in deformed condition. Reprinted from [86], with permission from Elsevier.

Bhuiyan and Alam [86] used the high-damping rubber bearing (HDRB) and combined SMA-based rubber bearing (SRB) shown in Figure 36 for reducing the ground acceleration of continuous highway bridges subjected to severe earthquake. Their analytical results indicated that the residual displacement of the deck is markedly reduced after earthquakes for SRB compared to HDRB bearings.

**Figure 37:**
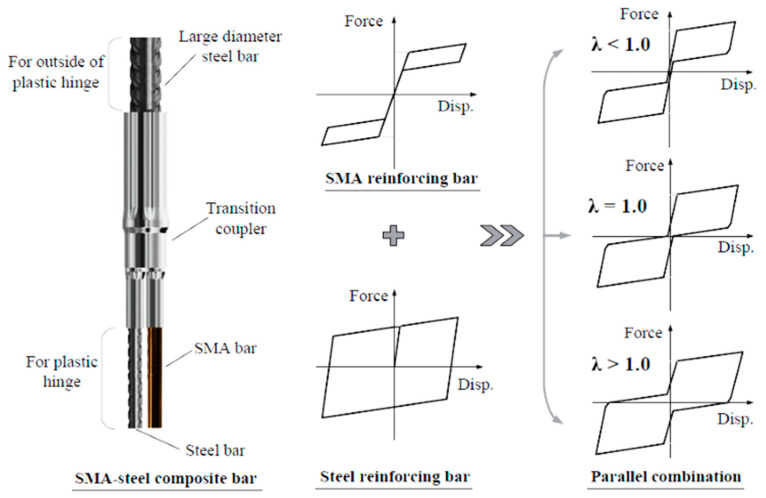
SMA–steel coupled reinforcing bar and its possible force–deformation relationships. Reprinted from [87], with permission from Elsevier.

**Figure 38:**
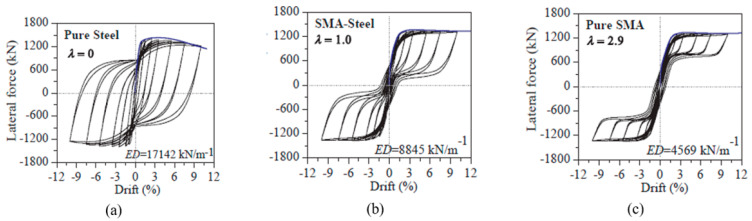
Hysteretic response of tested column–footing joint: (**a**) pure steel specimen; (**b**) SMA–steel specimen; (**c**) pure SMA specimen. Reprinted from [87], with permission from Elsevier.

Xiang, et al. [87] proposed a novel SMA–steel coupled system for piers of concrete bridges, to attain a balance between self-centering and energy dissipation capabilities, as shown in Figure 37. The proposed coupled system proved to be effective for enhancing the service life of the bridge against seismic excitations. They concluded that the bridge with λ = 1.0 for SMA–steel reinforcement is the most cost-effective in a life-cycle context as shown in Figure 38 (λ is defined as a ratio between the self-centering contribution and the energy dissipation contribution for a particular loading effect). Table 3 summarizes the applications of superelastic SMA in various bridge components.

#### 6.1.6. Beams

**Figure 39:**
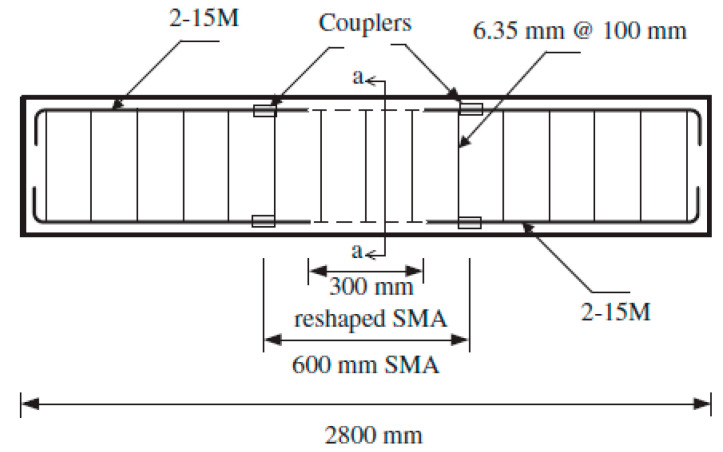
Reinforcement details of SMA beam. Reprinted from [89], with permission from Elsevier.

**Figure 40:**
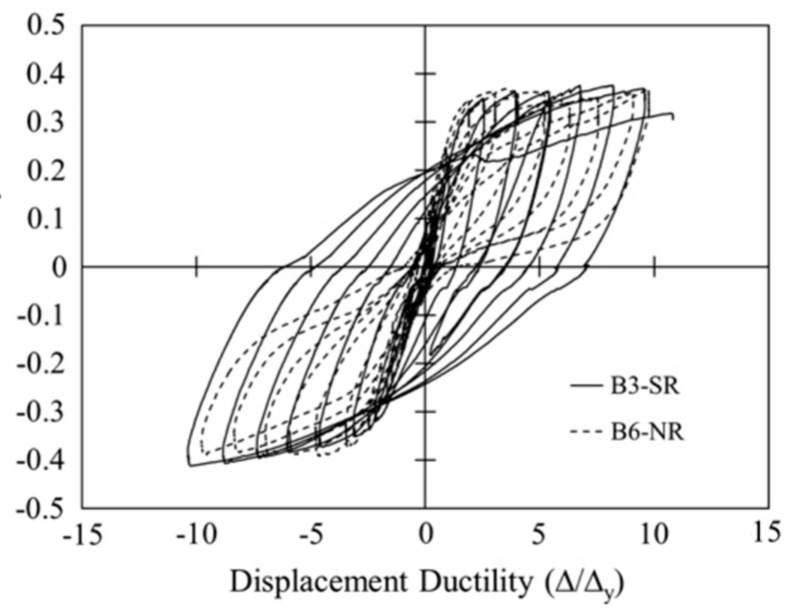
Normalized load–displacement ductility responses under reverse cyclic loading. Reprinted from [89], with permission from Elsevier.

Abdulridha et al. [89] investigated the flexural performance of concrete beams reinforced with SMA bars or conventional steel bars in the constant-moment zone under reversed cyclic loading, as shown in Figure 39. The experimental test results (Figure 40) demonstrated the superior ability of the SMA beams to recover the inelastic displacements, as well as sustain displacement ductility and strength compared to RC beams. In contrast, the crack spacing and widths were larger in case of the SMA beams compared to RC beams; however, the crack openings were recovered upon removal of load. Moreover, the SMA beams dissipated 54% of the energy dissipated by RC beams.

### 6.2. Retrofitting Applications

Smart memory alloys (SMAs) have great potential to be used for repair/retrofit of new/existing structures under static gravity loads or lateral cyclic loads simulating earthquakes. The recent retrofitting applications of SMAs are discussed in this section.

#### 6.2.1. Retrofitting Applications under Static Gravity Loads

**Figure 41:**
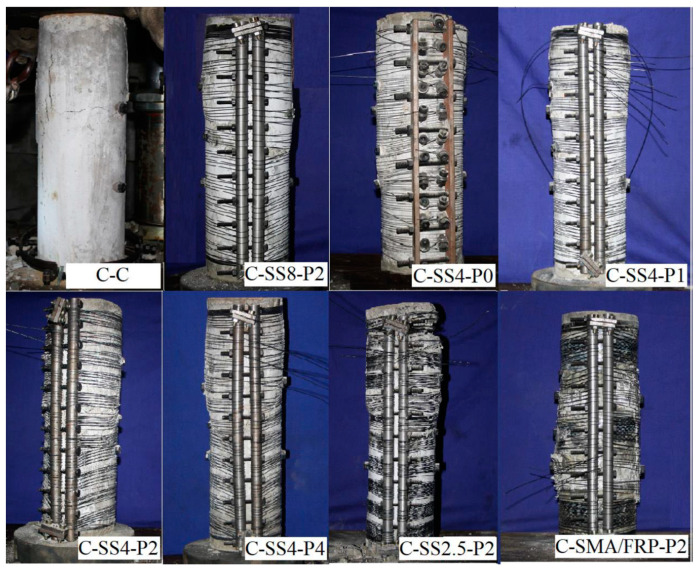
Failure patterns of reinforced concrete columns confined with superelastic SMA. Reprinted from [101], with permission from Creative Commons Attribution (CC BY) license.

**Figure 42:**
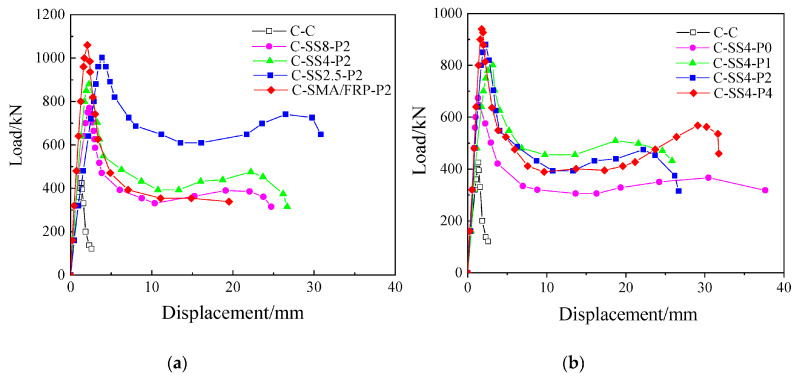
Load–displacement curves for RC columns (**a**) with different amounts of SMA wires, and (**b**) with different prestrain level of SMA wires. Reprinted from [101], with permission from Creative Commons Attribution (CC BY) license.

Hong et al. (2020) [101] investigated the effect of the amount of SMA and prestrain level on the compressive behavior of RC columns strengthened using superelastic NiTi wires. One more specimen was also tested using SMA and FRP bars together, as shown in Figure 41 The experimental test results indicated that the SMA wires enhanced the load-carrying capacity of the columns. Although SMA and SMA/FRP had comparable effects on the load capacity of concrete columns, columns reinforced with SMA had superior ductility, as shown in Figure 42a. At the same prestrain level, increasing the amount of SMA by decreasing the spacing from 8 mm to 2.5 mm resulted in increasing the load capacity from 80% to 135% compared to control columns. Meanwhile, at the same SMA amount, increasing the prestrain level from 0% to 4% led to an increase from 60% to 120% in the load capacity compared to control columns, as shown in Figure 42b.

#### 6.2.2. Retrofitting Applications under Lateral Cyclic Loading

Designing structures to withstand the vibrations induced by seismic actions is of primary concern for structural engineers worldwide. Different schemes have been proposed in the literature to enhance the seismic performance of existing non-seismically designed concrete frames using SMA. These methods are summarized in Table 4 and presented in detail in this section.

**Figure 43:**
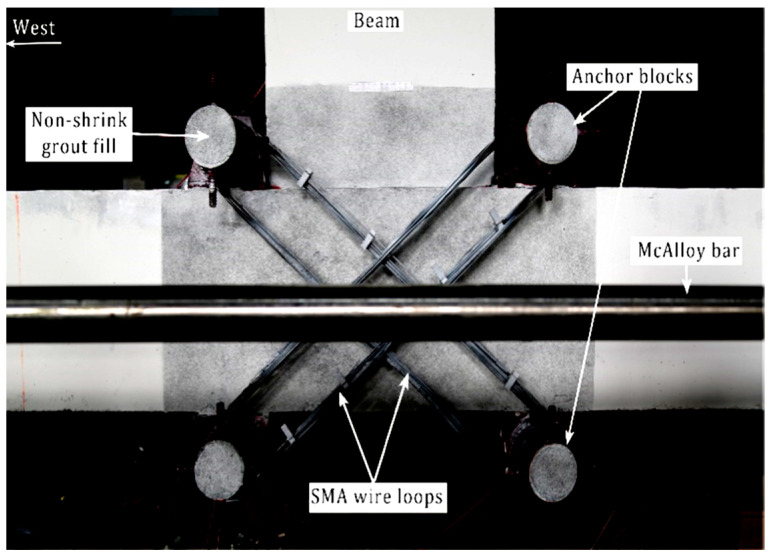
Scheme of retrofitted technique. Reprinted from [30], with permission from Elsevier.

**Figure 44:**
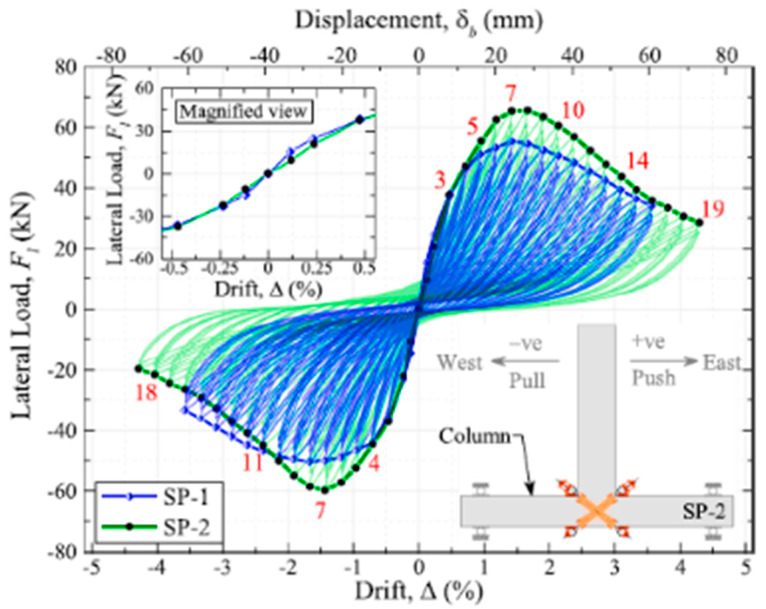
Lateral load vs. lateral drift of specimens. Reprinted from [30], with permission from Elsevier.

Suhail et al. [30] proposed using prestressed SMA loops for retrofitting non-seismically designed beam–column connections, as shown in Figure 43. Despite the noticeable increase in strength and ductility of the retrofitted joint as shown in Figure 44, the proposed method may not be the best for enhancing the ductility of beam–column connections.

**Figure 45:**
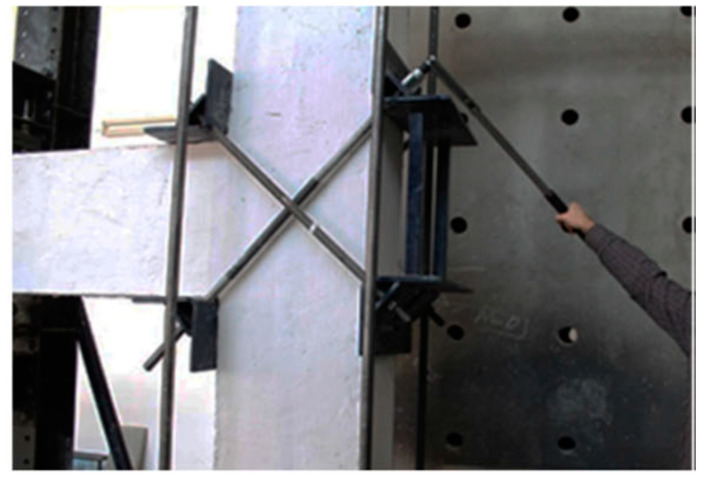
Scheme of retrofitted technique. Reprinted from [102], with permission from Springer Nature.

**Figure 46:**
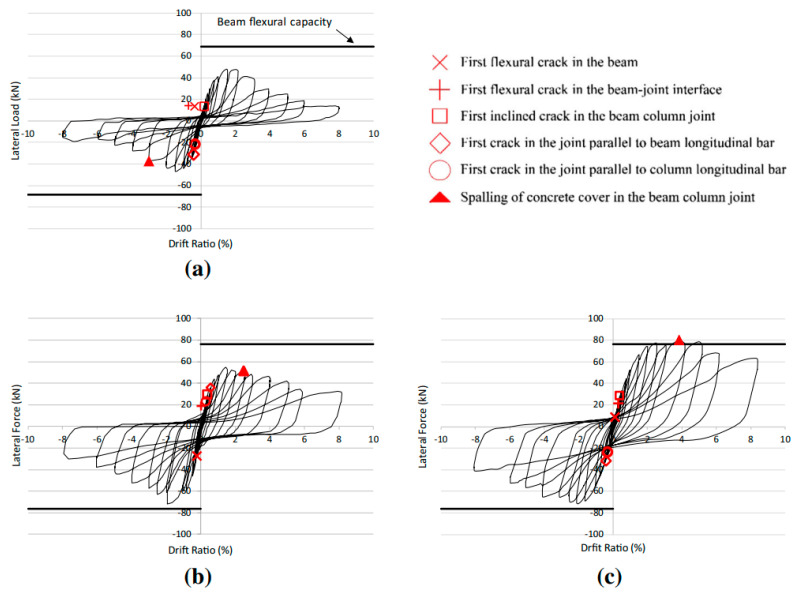
Hysteretic response of (**a**) control joint, (**b**) retrofitted joint using steel bars, and (**c**) retrofitted joint using SMA. Reprinted from [102], with permission from Springer Nature.

Yurdakul et al. [102] proposed an innovative post tensioning method for enhancing the seismic performance of non-seismically designed beam–column joints. This method comprised testing three full-scale joint specimens that are strengthened by post-tension rods as shown in Figure 45, under quasi-static cyclic loading up to 8% drift ratio. Two different post-tensioning superelastic NiTi alloy and steel bars were mounted diagonally on the joint, and then the required post-tension force was applied. The test results (Figure 46) indicated that the reference RC specimen exhibited a brittle failure with severe damage and excessive cracks mostly concentrated in the joint region. While the specimen retrofitted by the post-tensioned steel bar displayed some enhancement in strength, the overall performance was still dominated by shear failure in the joint region. On the other hand, the specimen retrofitted by post-tensioned SMA bars was capable of attaining an adequate performance with no observed strength degradation up to a drift ratio of 5% in both positive and negative load directions. Moreover, the test results proved that post-tensioning SMA bars with an applied force of 75% of their strength capacity played an important role in enhancing the retrofitting effectiveness of SMA without yielding until the last cycles.

### 6.3. Self-Healing Applications

Concretes reinforced with Nitinol (NiTi) SMA wires exhibit superior performance compared to concretes reinforced with steel wires. Their potential contribution to self-healing mechanism of concrete cracks is based on two approaches: external heating and superelastic properties of the alloy. In the first approach, with the initiation of cracks in concrete, the NiTi SMA wires are heated through an external power source, causing them to return to their original austenitic phase and contract. This contraction generates a force that closes the cracks and prevents their propagation, thus recovering the original shape and strength of cracked concrete. In the second approach, SMA wires are embedded in the concrete in a pretensioned state. When a crack forms, the wires are able to undertake enormous strains without causing permanent deformation. The strains of the SMA wires generate a force that closes the crack and restores the integrity of the cracked concrete. Kuang and Ou [29] used SMA wires to enhance the self-restoration ability of RC beams under a three-point bending test. The experimental test results indicated that the RC beams strengthened with SMA wires were able to return the deflected beam to its original shape before loading. Furthermore, healing of the cracks that occurred on the tension face of the beam was observed because of the recovery forces of SMA wires.

### 6.4. Fire Protection Applications

**Figure 47:**
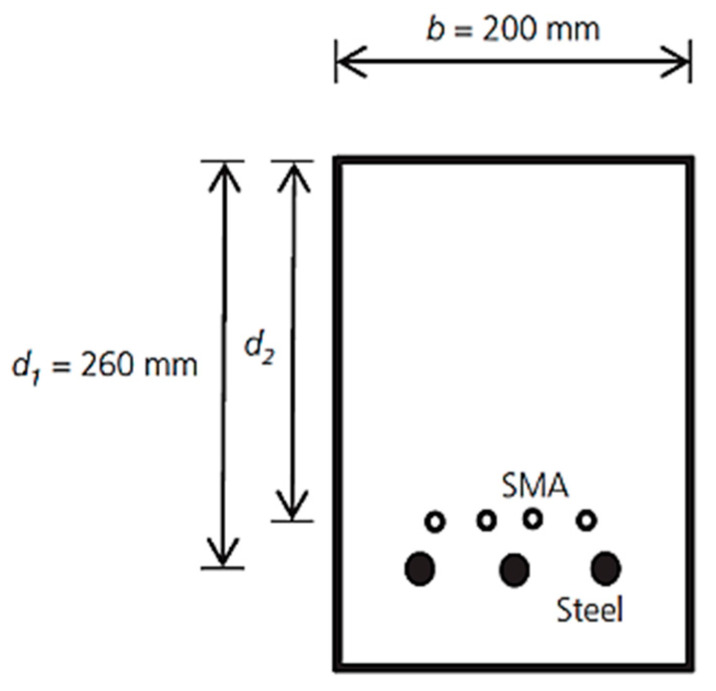
Cross-section of steel–SMA RC beam Reprinted from [103], with permission from Thomas Telford Limited.

**Figure 48:**
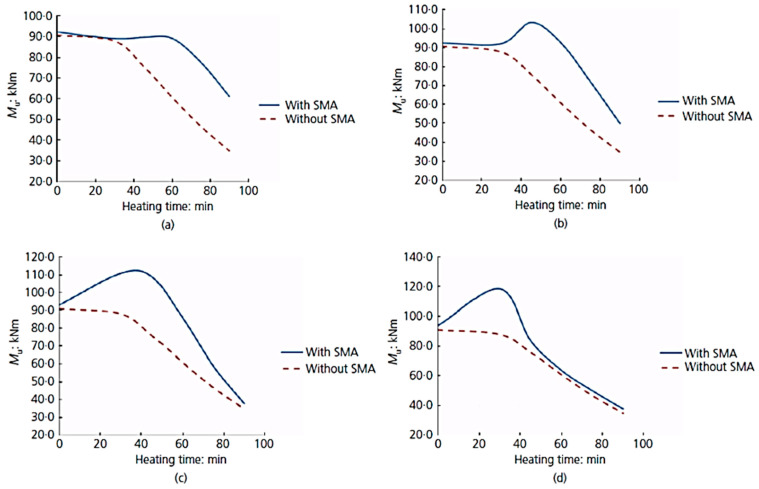
Effect of changing the SMA bar diameter on moment capacity: (**a**) d_2_ = 220 mm; (**b**) d_2_ = 230 mm; (**c**) d_2_ = 240 mm; (**d**) d_2_ = 260 mm. Reprinted from [103], with permission from Thomas Telford Limited.

Wong and Liu [103] proposed an innovative technique for enhancing the fire resistance of reinforced concrete (RC) beams. This technique is based on using hybrid reinforcement of steel and SMA bars in the tension region of the beam, as shown in Figure 47. The experimental test results (Figure 48) indicated that the bending moment capacities for various distances of SMA from the extreme compression fiber of the beam (d_2_) were enhanced significantly with the increase in the heating time in minutes. It can be observed that, for the beams reinforced with hybrid steel–SMA bars with the largest axis distance of SMA (d_2_ = 260 mm), the moment capacity was 75% more compared to beams without SMA, losing about 30% of their moment at the end of 90 min heating time.

Jia et al. [104] proposed a novel method for enhancing the fire resistance of prestressed concrete beams SMA bars and prestressed steel. This method is based on using SMA bars to compensate for the loss of prestressing force in prestressed steel bars upon exposure to fire. At elevated temperatures, the modulus and strength of SMA bars rise and, thus, induce a stress that can minimize the reduction in the mechanical properties of the steel bars. Although the flexural capacity and stiffness of the hybrid prestressed steel–SMA beam at elevated temperatures were lower than those of the ordinary prestressed beam at room temperature, the hybrid prestressed beam still maintained an acceptable performance that met the fire resistance requirements.

## 7. Conclusions

Shape memory alloys (often referred as smart materials) have great potential for enhancing the performance of civil engineering systems. The distinctive features of nickel-based superelastic shape memory alloys (NiTi SMAs) are extremely beneficial for the design, construction, and retrofit of RC structures. To foster the applications of SMA in the building industry, researchers should make their new discoveries compatible with existing design practices and prepare simple guidelines on SMA use for concrete practitioners. This paper presents a critical review of the applications of Nitinol (NiTi) SMAs in reinforced concrete structures over the last two decades. Since superelastic, SE SMA-based systems have numerous properties superior to SME SMAs, the present article focused more on the applications of SE Nitinol SMAs in civil infrastructure because of their simplicity to use and no need for a heat source to trigger their superelasticity. The distinctive features of SMA including the superelastic effect (SE) and the shape memory effect (SME) were discussed first. Then, the mechanical properties of NiTiSMA and their dependence on loading conditions, strain rate, and temperature were briefly examined, followed by a concise overview of SMA performance under corrosive environments and elevated temperatures. The most recent applications of NiTiSMA were discussed at length including seismic strengthening of footing–column connections and self-centering of beam–column joints, vibration control of bridges, prestressing concrete beams, shear walls, SMA fiber-reinforced concrete, self-healing of concrete cracks, and fire protection of concrete structures. To optimize the use of NiTi-based SMA for enhancing the seismic performance of RC structures, future studies should be directed toward innovative hybrid designs of SMA (bars, wires, and plates) and low-carbon steel reinforcement (bars, wires, plates, and meshes).

## 8. Recommendations and Future Opportunities

On the basis of this review, the following aspects need to be addressed adequately before SMAs can be further implemented in civil infrastructures:

- Bond behavior of SMA bars with concrete.
- Optimal design of concrete structures using SMAs to attain a balance between self-centering and energy dissipation capabilities.
- New processing technologies for manufacturing SMAs at lower costs.
- Hybridization of SMA with reinforcing steel to achieve optimal performance.
- More studies on the properties of coupled SMA–steel reinforcement system under corrosive environment.
- The concept of using SMA fibers for prestressing cementitious materials in civil engineering.
- New guidelines should be established to identify the amount of SMA needed for enhancing the performance of RC structures.
- The numerical modeling of SMA concrete structures should be linked to laboratory experiments to foster their applications in building industry.

## Figures and Tables

**Table 1:** Summary of the SMA properties used in the literature for concrete applications.

Element Type	Type of SMA	Ultimate Stress (MPa)	Yield Stress (MPa)	Recovery Stress (MPa)	Recovery Strain	Elastic Modulus (GPa)	Reference
Fiber/wire	NiTi	915	350	130	6%	31	[27]
		1150	550	200	8%	75	[28]
		920	560	230	7%	60	[29]
	NiTiNb	1059	635	550	8%	83	[30]
		1030	580	460	5%	56	[31]
Bar	NiTi	700	485	260	6%	55	[32]
		NR *	401	220	6%	62	[33]
		752	549	140	7%	54	[34]
		1068	380	190	6%	38	[35]
		830	480	250	7%	36	[36]

* NR: not recorded.

**Table 2:** Summary of the previous publications in self-centering application of SMAs in concrete structures.

Number	Structural Member *	Technique	Residual Displacement **	Energy Dissipation **	Load Capacity **	Study Method	Reference
1	Beam	SMA fiber concrete	79% decrease	22% decrease	8% decrease	Experimental	[28]
2	CFJ	Strengthening using external SMA bar at plastic hinge	58% increase	39% increase	72% decrease	=	[34]
3	CFJ	SMA bars at the plastic hinge using normal concrete	100% decrease	70% decrease	8% decrease	Numerical	[84]
4	CFJ	SMA bars at the plastic hinge using ultra high performance concrete	100% decrease	56% decrease	30% increase	=	[84]
5	CFJ	Strengthening using external SMA bar at plastic hinge and concrete jacketing	25% decrease	88% increase	183% increase	=	[85]
6	CFJ	Strengthening using external SMA bar at plastic hinge and ECC jacketing	40% decrease	110% increase	230% increase	=	[85]
7	BCJ	SMA bar at plastic hinge	75% decrease	37% decrease	3% increase	Experimental	[33]
8	BCJ	SMA bar at plastic hinge with slots	86% decrease	43% decrease	4% decrease	=	[32]
9	Shear wall	SMA bars at the plastic hinge	42% decrease	21% decrease	15% decrease	=	[35]
10	Shear wall	SMA bars at the plastic hinge with steel angle for precast wall	87% decrease	17% decrease	10% increase	Numerical	[36]
11	Bridge	NiTi SMA rubber bearings	84% decrease	30% increase	20% increase	=	[86]
12	Bridge	SMA bars at the plastic hinge near footing	88% decrease	73% decrease	7% decrease	=	[87]
13	Bridge	SMA–steel bars at the plastic hinge near footing	75% decrease	48% decrease	7% decrease	=	[87]

* BCJ: beam–column joint, CFJ: column–footing joint; ** results of SMA specimen compared to control steel specimen.

**Table 3:** Summary of previous publications on the use of NiTi SMAs for bridges in concrete structures.

#	SMA Element	Study Type	Application	Type of Loading	Technique	Findings	Reference
1	Washer	Experimental	Pier	Reversed cyclic	SMA-washer-based piers	The novel system showed low damage, negligible residual deformation, and protection against over-rocking.	[92]
2	Wire	Experimental	Damper	Dynamic by shake table	SMA damper for cable-stayed bridges between the tower and the deck	SMA damper reduced the tower accelerations, relative displacements, and the bending moments.	[93]
3	Cable	Experimental	Girder	Dynamic by shake table	SMA restrainer cables in-span hinges of box girder	SMA restrainer cables had slight residual strain with little strength and stiffness degradation after repeated loading	[94]
4	Bar	Experimental	Pier	Reversed cyclic	SMA bars and engineered cementitious composites (ECCs) at plastic hinge	The reduction in residual displacement was 83% when a combination of SMA bars and ECC was used in the plastic hinge zone, while the reduction was only 67% for conventional concrete and SMA.	[95]
5	Bar	Experimental	Pier	Dynamic by shake table	SMA bars and engineered cementitious composites (ECC) at plastic hinge	SMA minimized residual displacement, and the hybrid use of ECC and SMA was found to reduce significnatly the earthquake damage.	[96]
6	Bar	Numerical	Pier	Reversed cyclic	post-tensioned precast segmental bridge piers using SMA bars	SMA bars provided self-centering capability, increased the hysteretic energy dissipation, and achieved high ductile behavior.	[97]
7	Bar	Numerical	Pier	Reversed cyclic	SMA bars at plastic hinge	The SMA RC bridge pier showed superior performance to bridge piers reinforced with steel bars alone.	[98]
8	Wire	Numerical	Isolation device	Reversed cyclic	SMA-based rubber bearing	Residual displacement of the deck was reduced after moderate and strong earthquakes. Pier displacements were smaller, while deck displacement, bearing displacement, and deck acceleration were significantly larger.	[86]
9	Cable	Numerical	Restrainer	Cyclic	SMA-based restrainer	SMA devices were able to eliminate residual joint openings.	[99]
10	Bar	Numerical	Isolation device	Reversed cyclic	Laminated rubber bearing with a device made of SMA	The SMA isolation system had self-centering ability. For a medium-size earthquake, the SMA bars increased the damping capacity, whereas the SMA bars provided hysteretic damping and acted as a controlling device for a large earthquake.	[100]
11	Bar	Numerical	Pier	Reversed cyclic	SMA–steel bars at the plastic hinge near footing	The innovative SMA–steel coupled reinforcing bar showed similar effectiveness to the pure SMA bar in reducing the residual drift, while dissipating higher energy compared to pure SMA reinforcement.	[87]

**Table 4:** Summary of the previous publications in self-centering application of SMA in concrete structures.

#	Structural Member *	Technique	Load Capacity **	Energy Dissipation **	Ductility **	Study Method	Reference
1	BCJ	Diagonal prestressed SMA loops at the joint	18% increase	56% increase	5% decrease	Experimental	[30]
2	BCJ	Diagonal post-tension SMA bars	70% increase	160% increase	35% increase	Experimental	[102]

* BCJ: beam–column joint. ** The results of SMA specimen compared to control steel specimen

## Data Availability

The datasets generated and/or analyzed during the current study are available from the corresponding author upon reasonable request.
